# Tartrolon E, a secondary metabolite of a marine symbiotic bacterium, is a potent inhibitor of asexual and sexual *Plasmodium falciparum*

**DOI:** 10.1128/aac.00684-23

**Published:** 2024-01-09

**Authors:** Laura Chery-Karschney, Rapatbhorn Patrapuvich, Devaraja Gouda Mudeppa, Sreekanth Kokkonda, Rimi Chakrabarti, Patchara Sriwichai, Roberta M. O'Connor, Pradipsinh K. Rathod, John White

**Affiliations:** 1 Department of Chemistry, University of Washington, Seattle, Washington, USA; 2 Drug Research Unit for Malaria, Faculty of Tropical Medicine, Mahidol University, Bangkok, Thailand; 3 Department of Medicine, Goa Medical College and Hospital, Bambolim, Goa, India; 4 Department of Medical Entomology, Faculty of Tropical Medicine, Mahidol University, Bangkok, Thailand; 5 Department of Veterinary and Biomedical Sciences, College of Veterinary Medicine, University of Minnesota, Minneapolis, Minnesota, USA; The Children's Hospital of Philadelphia, Philadelphia, Pennsylvania, USA

**Keywords:** malaria, marine natural product, gametocidal, shipworm symbiont

## Abstract

Due to the spread of resistance to front-line artemisinin derivatives worldwide, there is a need for new antimalarials. Tartrolon E (TrtE), a secondary metabolite of a symbiotic bacterium of marine bivalve mollusks, is a promising antimalarial because it inhibits the growth of sexual and asexual blood stages of *Plasmodium falciparum* at sub-nanomolar levels. The potency of TrtE warrants further investigation into its mechanism of action, cytotoxicity, and ease with which parasites may evolve resistance to it.

## INTRODUCTION

Malaria is a major infectious disease caused by *Plasmodium spp*. parasites. Approximately 247 million cases and 619,000 deaths were attributed to malaria in 2021, mainly in sub-Saharan Africa (1). Of the five species that infect humans, *Plasmodium falciparum* is responsible for the vast majority of malaria-caused morbidity and mortality. The development and spread of resistance to derivatives of the front-line antimalarial artemisinin have intensified the need for discovery of novel inhibitors that target unique pathways (2, 3).

Natural products make up more than half of the FDA-approved drugs over the last 40 years and have long been an important source of and inspiration for antimicrobials due to their structural diversity (4, 5). Some of the most efficacious and widely used antibiotics are polyketides, a large, diverse class of natural products that include the tetracyclines (6) and the macrolides azithromycin and erythromycin (7), which all possess antimalarial properties. Two of the most successful antimalarials in history are the natural products quinine and artemisinin, which have complex mechanisms of action and require multifactorial processes for the acquisition of resistance (8). Looking forward, the ocean is an underexplored frontier for natural product discovery, and marine organisms have been shown to produce a wide variety of unique chemical scaffolds with biomedical potential (9).

**Fig 1:**
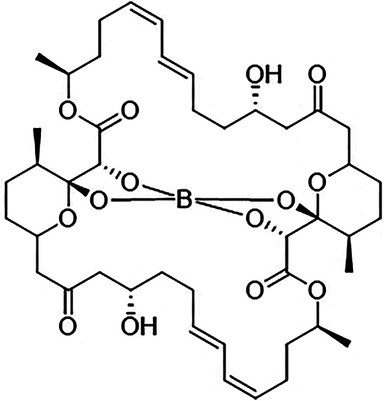
Tartrolon E. TrtE (C_44_H_64_BO_14_; molecular weight = 827.79) was isolated from a culture of the *T. turnerae* bacterium and purified as previously described (11) (purification method 2). The identity of TrtE was confirmed by high-resolution mass spectrometry electrospray ionization (HRMS-ESI) as well as ^1^H and ^13^C nuclear magnetic resonance (NMR) (data not shown).

Tartrolon E (TrtE) (Fig. 1) is a secondary metabolite macrolide polyketide with a central complexed tetraborate. It was isolated from *Teredinibacter turnerae*, an intracellular endosymbiotic gammaproteobacteria of marine wood-boring bivalve mollusks of the family *Teredinidae* (shipworms) (10). Recently, we showed the potent inhibition of a diverse range of apicomplexan parasites including *Plasmodium*, *Toxoplasma*, and *Cryptosporidium* by TrtE (11). This was consistent with the hypothesis that *T. turnerae* may produce TrtE to protect mollusks against gregarines, the most ancestral organism of the apicomplexan phylum (12). Here, we describe the bioactivity of TrtE on asexual and sexual blood stages of *Plasmodium falciparum*.

**Fig 2:**
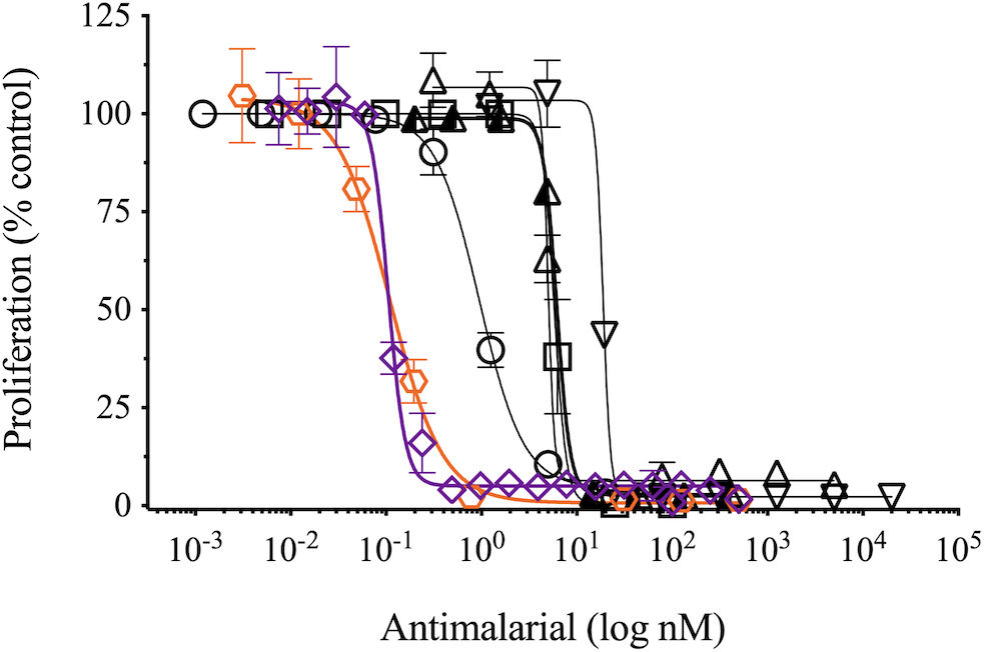
Inhibition by TrtE vs established and novel antimalarials. Standard, 72-hour inhibitor-response assays (14) were utilized to determine the 3D7 EC_50_s for tartrolon E (purple diamond, 105pm), dihydroartemisinin (orange hexagon, 129 pM), atovaquone (circle, 1 nM), DSM265 (triangle, 5 nM), artemisinin (half-filled triangle, 6 nM), chloroquine (square, 6 nM), and pyrimethamine (upside–down triangle, 19 nM).

Historically, the best antimalarials are highly potent, tolerable, and efficacious across multiple steps of the parasite life cycle (13). In parasite proliferation assays (14), TrtE exhibited sub-nanomolar potency against the asexual blood stages of *P. falciparum* (*Pf*) (3D7 EC_50_ = 105 pm), responsible for the clinical manifestations of malaria disease. *In vitro* inhibition of parasite growth by TrtE was approximately equivalent to that of dihydroartemisinin (DHA; 3D7 EC_50_ = 129 pm), the active metabolite of the front-line artemisinin (Fig. 2).

A diverse panel of *P. falciparum* clones, collected from four continents with varying genetic backgrounds and well-characterized susceptibilities to novel and existing antimalarials, was treated with TrtE and DHA in standard growth inhibition assays. All clones were inhibited by TrtE at sub-nanomolar concentrations, including two clones from Western Cambodia that were collected in a province with clinical artemisinin resistance and that display *in vitro* resistance to artemisinin in ring-stage survival assays (RSAs) (BEI, Didier Menard) (Table 1). The potency of TrtE is comparable to DHA across all parasite lines tested, with both compounds displaying slightly lower proliferation inhibition to the Cambodian cell lines. Based on these data, the preliminary indications are that the mechanism of action utilized by TrtE is orthogonal to those of all commonly used antimalarials except DHA. Additional studies are needed to determine whether there is any similarity in mechanism between TrtE and DHA.

**TABLE 1:** *Pf* clone-specific sensitivities to TrtE^*a*^

Cell line	Origin	EC50 (nM) (95% CI)		CQ	QN	MQ	CG	PYR	SDX	ART	ATO	DSM1
		TrtE	DHA									
IPC 5202	Battambang, Cambodia	0.81 (0.76–0.86)	0.94 (0.72–1.03)	R	R	R	R	R	R	R	S	S
IPC 3445	Palin, Cambodia	0.73 (0.69–0.79)	0.92 (0.91–1.10)	R	R	R	R	R	R	R	S	S
Dd2	SE Asia	0.28 (0.27–0.32)	0.14 (0.12–0.16)	R	R	R	R	R	R	S	S	S
GMC1	Goa, India	0.26 (0.23–0.30)	0.21 (0.19–0.24)	R	U	S	R	R	R	S	S	S
TM90C2A	Thailand	0.16 (0.14–0.17)	0.091 (0.086–0.097)	R	U	R	R	R	U	S	S	S
7G8	Brazil	0.18 (0.16–0.19)	0.095 (0.083–0.110)	R	R	S	R	R	U	S	S	S
V1/S	Vietnam	0.31 (0.29–0.33)	0.084 (0.072–0.090)	R	R	S	R	R	U	S	S	S
HB3	Honduras	0.13 (0.12–0.14)	0.16 (0.13–0.20)	S	S	S	S	R	S	S	S	S
NF54	Netherlands	0.20 (0.17–0.25)	0.15 (0.14–17)	S	S	S	S	S	R	S	S	S
3D7	Netherlands	0.16 (0.14–0.18)	0.11 (0.10–0.12)	S	S	S	S	S	R	S	S	S
D6	Sierra Leone	0.14 (0.13–0.15)	0.17 (0.14–0.20)	S	S	S	S	S	S	S	S	S
D10 w/ yDHODH	Papua New Guinea	0.19 (0.17–0.21)	0.12 (0.11–0.13)	S	R	S	U	U	U	S	R	R

^*a*^ TrtE displays high potency against a broad set of parasite genetic backgrounds. R, *P. falciparum*-resistant as determined by EC50 or, for ART, ring-stage survival assay (RSA); S, sensitive; and U, undetermined. CQ, (chloroquine); QN (quinine); MQ, (mefloquine); CG, (cycloguanil); PYR, (pyrimethamine); SDX, (sulfadoxine); and ATO, (atovaquone). Parasite clones were obtained from BEI Resources except for GMC1, which was collected in Goa, India, by the MESA-ICEMR. Cambodian clones were reported as resistant to artemisinin by RSA (BEI, Didier Menard).

**Fig 3:**
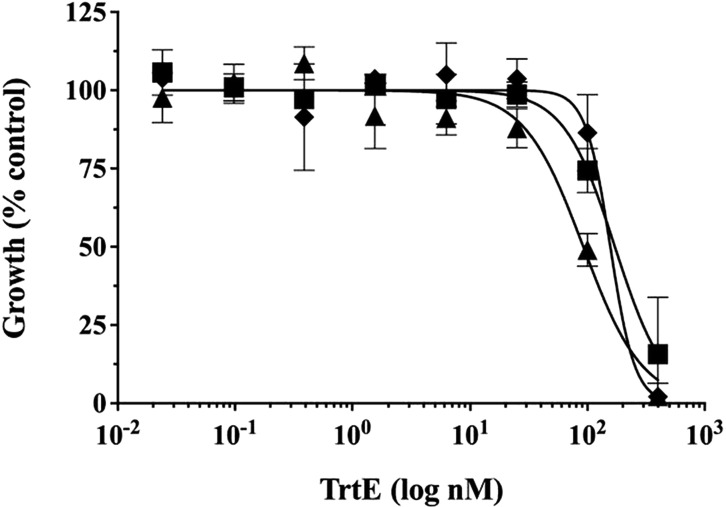
TrtE activity against gametocytes. Enriched, late-stage gametocytes were treated for 3 days with TrtE and then viability-tested with alamarBlue (Invitrogen) (15, 16). Three independent experiments (squares, triangles, and diamonds) determined the average EC_50_ to be 140 ± 42 nM.

**Fig 4:**
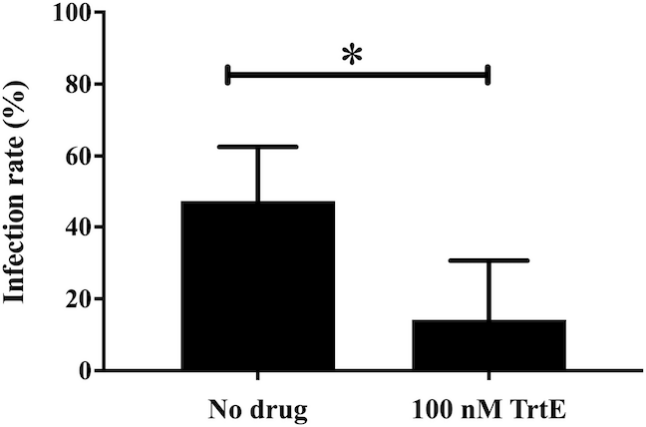
TrtE inhibition of oocyst development in mosquitoes. Late-stage *Pf* NF54 gametocytes were incubated with 100 nM TrtE, the approximate gametocidal EC_50_, or left untreated. Following incubation and development to stage V, the cultures were fed to female *Anopheles dirus* mosquitoes using standard membrane feeding techniques. After 9 days, the mosquitoes were dissected, and oocysts were stained and counted. The infection rate was measured as the ratio of mosquitoes with one or more oocysts to the number of dissected mosquitoes. ^*^P < 0.05.

Gametocytes of *Pf* NF54, the competent gametocyte-producing clone, were treated with TrtE as previously described (15, 16). In three independent experiments, TrtE inhibited the progression of *Pf* NF54 late-stage gametocytes (stage III to stage V) with an average EC_50_ of 140 nM, which is superior to the gametocytocidal antimalarials pyronaridine (4.26 µM) and primaquine (>40 µM) (17) (Fig. 3). To validate the *in vitro* gametocytocidal activity of TrtE, mosquito infectivity assays were conducted. In four independent experiments, treatment with 100 nM TrtE, the approximate gametocidal EC_50_, significantly inhibited mosquito infection by *P. falciparum* (*P* = 0.016) (Fig. 4). Oocyst development in mosquitoes represents a significant bottleneck in the life cycle of *Plasmodium spp*. where transmission can be efficiently interrupted (18).

In preliminary experiments to evaluate cytotoxicity to mammalian cells, the murine leukemia L1210 cell line and the human liver cell line HepG2 were grown under identical conditions alongside *Pf* 3D7. These were treated with TrtE, the chemotherapeutic methotrexate, and DSM265, an inhibitor of parasite dihydroorotate dehydrogenase (19). *In vitro* cytotoxicity assays demonstrated that TrtE inhibits L1210 growth at sub-micromolar concentrations, though the compound was 2,000-fold more toxic to parasites (Table 2). HepG2 was approximately threefold more sensitive to TrtE compared to L1210 cells (HepG2 EC_50_ 0.07 nM vs L1210 EC_50_ 0.23 nM). Previous toxicity selectivity indices (1302–2633) were comparable (11). A wide range of cellular effects of TrtE is consistent with other macrolides. In the future, selectivity may be improved with chemical modification.

**TABLE 2:** Cellular sensitivity of TrtE^*a*^

Compound	EC_50_ (µM) (95% CI)			Selectivity
	3D7	HepG2	L1210	
Tartrolon E	0.00016 (0.00014–0.00018)	0.071 (0.063–0.081)	0.23 (0.21–0.25)	444; 1,438
DSM265	0.005 (0.0038–0.006)	9.9 (8.2–12.0)	>20	1,980; >4,000
Methotrexate	0.047 (0.041–0.054)	>1	0.012 (0.011–0.013)	0.26; 21.3

^*a*^ The selectivity index (EC50 of HepG2 or L1210/EC50 3D7) of TrtE was compared to the antimalarial DSM265 (phase IIa clinical trial) and the FDA-approved anti-arthritic, anti-cancer drug methotrexate.

TrtE may be considered for further development because of its high potency across multiple stages of the malaria parasite life cycle. Preliminary *in vitro* single-step selection experiments indicate that pressure from TrtE presents high hurdles to resistance for asexual parasites (data not shown). TrtE is a complex molecule that requires significant effort to extract and would be challenging to synthesize; however, further investigation may disclose a minimum scaffold responsible for the activity. In addition to further defining windows of parasite–host cytotoxicity, high priorities include the characterization of the kinetics of TrtE-mediated killing and identification of the cellular target(s) of TrtE using forward genetic approaches.
